# Epidemiology of Parkinson’s disease – Global burden of disease research from 1990 to 2021 and future trend predictions

**DOI:** 10.1016/j.prdoa.2026.100421

**Published:** 2026-01-09

**Authors:** Sen Wang, Yujie Che, Yiqun Lin, Yukang Zhang, Wen He, Wei Zhang

**Affiliations:** Department of Ultrasound, Beijing Tiantan Hospital, Capital Medical University, Beijing 100050, China

**Keywords:** Parkinson’s disease, Global burden of disease, Forecasting, Disability-adjusted life years, Incidence, Prevalence

## Abstract

**Background:**

Parkinson’s disease (PD), a common neurodegenerative disorder, severely affects patients’ quality of life. This study aims to update the assessment of PD’s prevalence, incidence, mortality, and disability–adjusted life years (DALYs) from 1990 to 2021. Analyses were conducted at Socio-demographic Index (SDI), global, regional, and national levels, stratified by gender and age.

**Methods:**

PD data from the Global Burden of Disease (GBD) 2021 database was extracted. Trends in age-standardized prevalence rate (ASPR), age-standardized incidence rate (ASIR), age-standardized mortality rate (ASMR), and age-standardized DALY rate (ASDR) of PD were assessed, and estimated annual percentage change (EAPC) was calculated during the study period. Analyses were done by gender, age, GBD region, and SDI quintiles, using R statistical software for analyses and mapping.

**Results:**

In 2021, the global burden of PD remained substantial. The number of prevalent PD cases grew from 3,148,394.56 in 1990 to 11,767,271.97 in 2021 (a 2.74-fold increase). Incident cases rose from 417,134.69 to 1,335,142.12 (a 2.20-fold increase). Males exhibited higher prevalence, incidence, and mortality rates than females across almost all age groups. High-SDI regions had higher ASPR, ASIR, and ASDR values than low-SDI regions. Countries and regions in Asia, particularly China and Japan, exhibited among the highest ASPR and ASIR globally. Projections indicate global ASPR and ASIR will continue to rise substantially over the next three decades (to 2050), with males exhibiting a faster rate of increase than females, while the ASMR is predicted to remain essentially stable.

**Conclusion:**

This study systematically delineates the epidemiological landscape, temporal trends, and burden profiles of Parkinson’s disease across global, regional, gender-stratified, and age-specific populations, providing an evidence-based framework to guide precision prevention strategies and optimize healthcare resource prioritization.

## Highlights

• Global Parkinson's burden was analyzed using GBD 2021 data from 1990 to 2021.
• Trends were stratified by region, gender, age, and Socio-demographic Index.
• ARIMA and ES models projected the global disease burden for the next 30 years.
• Results provide a framework for precise prevention and resource allocation.

## 1 Introduction

Parkinson's disease (PD), a prevalent neurodegenerative disorder, is pathologically characterized by progressive degeneration and depletion of dopaminergic neurons in the substantia nigra pars compacta. This neurodegeneration results in striatal dopamine depletion, manifesting as a constellation of motor manifestations (e.g., resting tremor and bradykinesia) and non-motor sequelae (including cognitive decline and sleep architecture disturbances), collectively and profoundly compromising patients' functional independence and quality of life [1], [2]. Data from the Global Burden of Disease (GBD) 2019 estimates indicate that approximately 8.5 million people worldwide have PD [3]. Amidst demographic transitions toward aging populations and the enduring impacts of the COVID-19 pandemic, PD has exhibited a sustained upward trajectory in incidence rates, accompanied by marked transformations in its epidemiological profile. This emerging global public health challenge is now commanding significant attention from the international health community [4], [5].

In the field of epidemiologic research on PD, GBD studies play a crucial role by systematically assessing the global burden of numerous diseases [6]. There are significant differences in the prevalence of PD in different parts of the world. Between 1990 and 2011, for example the incidence of PD declined significantly in the Netherlands [7], although it increased dramatically in most other parts of the world [8], [9]. Current research on PD exhibits significant methodological limitations. A critical limitation lies in the predominant focus on isolated epidemiological dimensions—such as local prevalence rates or incidence trends in specific regions—while lacking robust, integrated analyses of PD's epidemiological characteristics and disease burden across geographical regions, gender groups, and age cohorts. This fragmented research paradigm impedes the development of holistic public health frameworks for PD management [8], [10], [11]. Therefore, there is an urgent need for comparable, consistent, and systematic analyses of PD burden and trends across regions and nations, coupled with predictive modeling of its global epidemiological trajectory. Such comprehensive assessments are critical for informing targeted public health interventions and optimizing resource allocation strategies.

To address this critical research gap, this study leverages the GBD 2021 database to systematically assess the global epidemiology and burden of PD—including prevalence, incidence, mortality, and disability-adjusted life years (DALYs)—across Socio-demographic Index (SDI) quintiles, 21 GBD regions, 204 countries/territories, and stratified by sex and age groups from 1990 to 2021. We further project burden estimates to 2050. Specifically, this study aims to answer: How do PD burden metrics (age-standardized prevalence rate, ASPR; age-standardized incidence rate, ASIR; age-standardized mortality rate, ASMR; age-standardized DALY rate, ASDR) vary geographically and across sex/age groups in 2021? How have ASPR, ASIR, ASMR, and ASDR evolved globally and regionally over the past three decades (1990–2021), as measured by estimated annual percentage change (EAPC)? How does SDI relate to burden? How are global ASPR, ASIR, ASMR, and ASDR expected to change from 2021 to 2050, and are there sex-specific patterns?

## 2 Methods

### 2.1 Data acquisition

This study sourced PD data from the GBD 2021 database, which encompasses the latest epidemiological estimates of 371 diseases and injuries across 21 GBD-defined regions and 204 countries/territories from 1990 to 2021. This comprehensive repository provides a standardized evidence base for comparative disease burden research, integrating multidimensional metrics [3]. All of these data are freely available on the Global Health Data Exchange (https://vizhub.healthdata.org/gbd-results/). The core tool for generating GBD epidemiological estimates for non-fatal conditions, such as PD, is the Bayesian *meta*-regression model, specifically the DisMod-MR V.2.1 version. This model serves as a critical component of the GBD methodological framework and is specifically designed for integrating, adjusting, and harmonizing heterogeneous epidemiological data. These data originate from diverse sources (including scientific literature, disease registries, and population surveys) and exhibit varying quality. This model enables the generation of spatiotemporally continuous and age-specific estimates of disease parameters (such as incidence, prevalence, and mortality) [12], [13], [14]. However, the quality of the model's output is highly contingent on the quality, representativeness, and coverage of the input data. In regions with extremely scarce data or systematic measurement biases, the uncertainty of its estimates increases substantially (as reflected in the 95% uncertainty intervals (UIs) provided by the GBD) [15]. For PD, the GBD research team input all relevant globally collated epidemiological data into the DisMod-MR V.2.1 model. Utilizing covariates (such as geographic location and time) and a hierarchical prior structure, the model integrates, adjusts, and harmonizes these raw data. By executing a complex Markov chain Monte Carlo (MCMC) algorithm, the model ultimately generated key parameter estimates for PD—including incidence, prevalence, and mortality—stratified by age and sex at the global, regional, and national levels for the period 1990–2021, along with their corresponding 95% UIs [3], [6]. These model-generated parameter estimates serve as the foundational input data for the subsequent calculation of age-standardized rates, trend analysis, and projections in this study.

### 2.2 SDI and GBD regional classifications

This study utilized the SDI and its country classifications directly from the GBD database. All countries or regions were stratified according to the five predefined SDI quintiles (Low, Low-middle, Middle, High-middle, and High) as established by GBD for analytical purposes [6]. The GBD 2021 study provides results not only for geographically defined regions and countries but also aggregates data according to several analytical groupings. In this study, when presenting results (such as in cluster analysis), we utilized these pre-defined GBD groupings which are available for download from the GBD database. These include: World Bank Income Levels: Classifications based on gross national income per capita. World Bank Regions: Geographic groupings used by the World Bank. Health System Groupings: Classifications based on the performance and characteristics of health systems.

### 2.3 EAPC

In this study, we calculated the EAPC by fitting a time-series regression model to assess the temporal trends of the age-standardized rates (ASRs) [4]. Specifically, in the equation y = α + βx + ε, Y refers to ln (ASR), X refers to calendar year, α refers to the intercept, β represents the annual change in ln (ASR), and ε represents the error term. This gives EAPC = 100 × (exp(β) − 1). Furthermore, the 95% confidence intervals (CIs) for the EAPC were calculated using the fitted model. An upward trend in age-standardized indicators is identified when both the EAPC value and the lower bound of its 95% CI exceed zero. Conversely, a downward trend is inferred when the EAPC value and the upper bound of its 95% CI are both below zero. In cases where the 95% CI encompasses zero, the trend is classified as stable, indicating no statistically significant directional change.

### 2.4 Autoregressive integrated moving average model (ARIMA) and exponential smoothing (ES) models

We selected the ARIMA model because it is a standard and robust tool for analyzing time-series data exhibiting temporal trends and autocorrelation properties. ARIMA models are widely applied in epidemiological forecasting due to their strong capacity to capture inherent structures within time-series data, such as trends and seasonality. They are particularly well-suited for extrapolating future trends based on historical patterns. Their flexibility in adapting to diverse time-series characteristics is achieved through model order parameters (p, d, q), while goodness-of-fit can be evaluated using information criteria (AIC, BIC). The resulting forecasts offer good interpretability and are extensively utilized in epidemiological burden projection studies. The ES model is recognized for its simplicity, robustness, and relatively lower data requirements. It predicts future values by assigning greater weight to recent observations, performing well with time-series data exhibiting stable trends but no pronounced complex patterns. Employing both ARIMA and ES models, which operate on distinct principles, facilitates cross-validation of result robustness. Convergent prediction trends derived from both models enhance confidence in future burden assessments. Conversely, divergent results necessitate cautious interpretation or exploration of model uncertainty sources. This dual-model strategy inherently mitigates potential limitations associated with reliance on a single model.

### 2.5 Statistical analysis

All statistical analyses were conducted within the R programming environment (version 4.3.1) under a rigorous methodological framework encompassing data cleaning, computational modeling, and graphical representation. Data visualization was implemented using the ggplot2 package, with predictive analytics conducted through the forecast package. As this study utilized publicly available datasets, the Institutional Review Board at Beijing Tiantan Hospital, Capital Medical University granted ethical exemption. All analytical procedures involving the GBD database strictly adhered to the Guidelines for Accurate and Transparent Health Estimates Reporting (GATHER), following cross-sectional research protocols outlined in the guidelines [15].

## 3 Results

### 3.1 Global level

The global burden of PD has increased between 1990 and 2021. The total number of cases of PD reached 11,767,271.97 in 2021 compared to 3,148,394.56 cases in 1990, representing a 2.74-fold increase over this period. Among them, the number of male patients was 6,438,639.86 cases and the number of female patients was 5,328,632.12 cases. In the same year, the number of new cases of PD worldwide amounted to 1,335,142.12 cases, an increase of about 2.20 times compared with 417,134.69 cases in 1990, and the number of deaths amounted to 388,194.26 cases, an increase of about 1.62 times compared with 148,067.94 cases in 1990 (Table 1).

**Table 1:** All-age cases and age-standardized prevalence, incidence, deaths, DALYs, YLDs and YLLs rates in the years of 1990 and 2021 for PD in global.

	2021			1990		
	All age cases			All age cases		
	Total	Male	Female	Total	Male	Female
Prevalence	11767271.97	6438639.86	5328632.12	3148394.56	1558967.49	1589427.07
Incidence	1335142.12	762142.24	572999.88	417134.69	222778.35	194356.34
Deaths	388194.26	219953.01	168241.24	148067.94	78276.19	69791.75
DALYs	7471821.42	4271994.64	3199826.78	2854049.82	1528363.66	1325686.16
YLDs	1670296.23	923231.96	747064.28	450235.6	224986.42	225249.18
YLLs	5801525.19	3348762.68	2452762.51	2403814.22	1303377.23	1100436.99

	ASRs per 100,000 people			ASRs per 100,000 people		
	Total	Male	Female	Total	Male	Female
Prevalence	138.63	168.24	114.47	88.28	99.22	77.09
Incidence	15.63	19.72	12.36	11.23	14.13	9.29
Deaths	4.81	6.57	3.59	4.62	6.28	3.62
DALYs	89.59	117.47	68.56	81.48	105.99	65.37
YLDs	19.62	23.95	16.07	12.22	14.09	10.87
YLLs	69.97	93.52	52.49	69.26	91.90	54.50

DALYs, disability-adjusted life years; YLDs, years lived with disability; YLLs, years of life lost; ASRs, age-standardized rates.

**Fig. 1:**
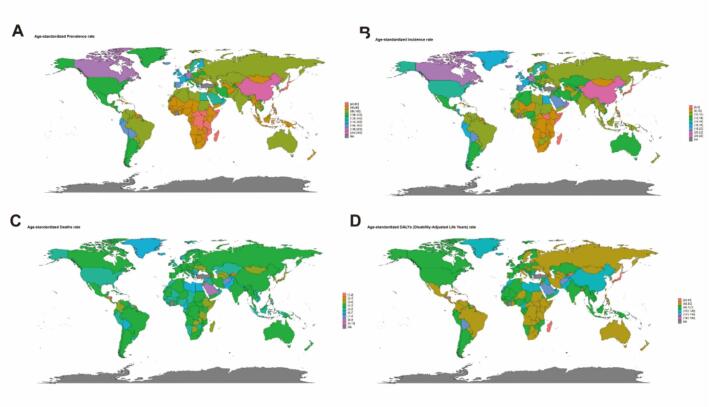
Spatial distribution of the age-standardized global burden of disease for PD in 2021. (A) ASPR (age-standardized prevalence rate). (B) ASIR (age-standardized incidence rate). (C) ASMR (age-standardized mortality rate). (D) ASDR (age-standardized DALY rate). DALYs, disability-adjusted life years.

**Fig. 2:**
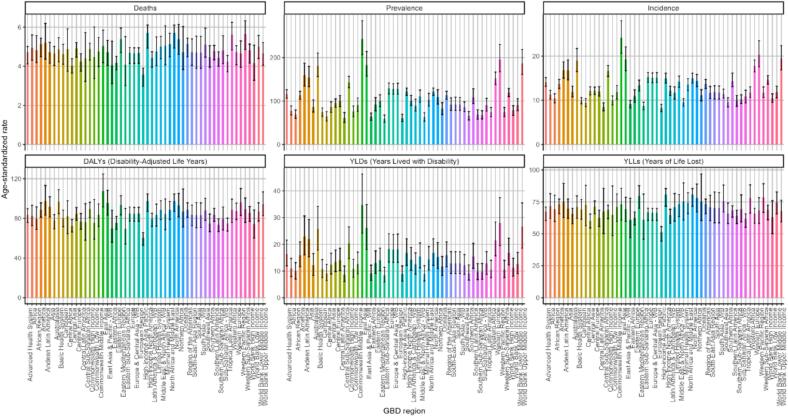
Age-standardized regional distribution of PD GBD in 2021. PD, parkinson's disease.

Analysis of Fig. 1, Fig. 2 shows that the global burden of disease for Parkinson's disease varies significantly across the 204 countries and territories of the world. countries and regions with high ASPR and ASIR are China and Japan in Asia and Canada in North America, in contrast to the relatively low burden of Parkinson's disease in most countries and regions in Africa. However, it is interesting to note that despite the significant differences in the global burden of Parkinson's disease, the ASMR is not very different. However despite the significant differences in the global burden of PD, the ASMR is not very different between countries and regions with high and low ASPR and ASIR.

Specifically, the ASPR for PD varied significantly between countries and regions, ranging from approximately 49.02 to 245.73 cases per 100,000 population. Among all countries and regions, China (245.73/100,000; 95% UI: 208.28–245.73), Israel (199.71/100,000; 95% UI: 166.21–240.28) and Canada (197.61/100,000; 95% UI: 184.67–209.27) had the highest ASPR (Fig. 1A, Table 2). Similarly, the ASIR for PD varied considerably between countries and regions, ranging from approximately 7.28 to 24.34 cases per 100,000 population. China (24.34/00,000; 95% UI: 20.67–28.30), Qatar (24.22/100,000; 95% UI: 21.16–27.95) and Israel (21.87/100,000; 95% UI: 18.51–26.12) had the highest ASIR (Fig. 1B, Table 2). Honduras ranked first in both ASMR and ASDR at 9.65 per 100,000; 95% UI: 7.76–11.65 and 157.67per 100,000; 95% UI: 131.01––188.02, respectively (Fig. 1C and 1D, Table 2). It is worth focusing on the fact that two of the three countries and regions with the highest ASPR and ASIR are located in Asia, namely China and Israel, yet these two countries do not have high ASMR.

**Table 2:** Top 10 countries and regions after age standardization in 2021.

ASPR		ASIR		ASMR		ASDR	
China	245.73 (245.73–208.28)	China	24.34 (28.30––20.67)	Honduras	9.65 (7.76,11.65)	Honduras	157.67 (131.01,188.02)
Israel	199.71 (240.28–166.21)	Qatar	24.22 (27.95––21.16)	Saint Kitts and Nevis	9.09 (7.98,9.93)	Saint Kitts and Nevis	143.73 (125.16,159.25)
Canada	197.61 (209.27–184.67)	Israel	21.87 (26.12–18.51)	Saudi Arabia	8.2 (6.78,10.10)	Saudi Arabia	138.08 (116.74,165.19)
Taiwan (Province of China)	194.47 (185.60,204.06)	Germany	21.53 (20.78,22.31)	Nauru	7.39 (5.35,9.44)	Nauru	132.58 (99.42,168.86)
Germany	186.45 (178.73,194.45)	Taiwan (Province of China)	21.29 (20.47,22.34)	Afghanistan	7.19 (5.44,9.19)	Afghanistan	126.51 (94.53,159.77)
Spain	177.82 (146.98,207.54)	Canada	21.05 (20.04,21.99	Guinea-Bissau	6.91 (5.43,8.30)	Bolivia (Plurinational State of)	120.78 (95.43,152.85)
Iceland	172.6 (140.72,207.73)	Oman	20.18(17.24,22.94)	Bahrain	6.8 (5.79,7.86)	Marshall Islands	116.75 (95.37,142.42)
Netherlands	168.81 (148.56,192.20)	Iceland	20.15 (17.31,23.40)	Montenegro	6.78 (5.74,7.80)	Greenland	116.37 (90.96,142.87)
Democratic People's Republic of Korea	161.47 (132.14,191.36)	Spain	19.97 (17.46,22.46)	Greenland	6.76 (5.12,8.64)	Qatar	115.82 (94.76,141.07)
Qatar	161.22 (128.85,198.10)	United Arab Emirates	19.56 (17.31,22.48)	Bolivia (Plurinational State of)	6.76( 5.19,8.77)	Guinea-Bissau	111.32 (87.91,131.01)

ASPR, age-standardized prevalence rate; ASIR, age-standardized incidence rate; ASMR, age-standardized mortality rate; ASDR, age-standardized DALY rate.

### 3.2 PD burden by age and gender

**Fig. 3:**
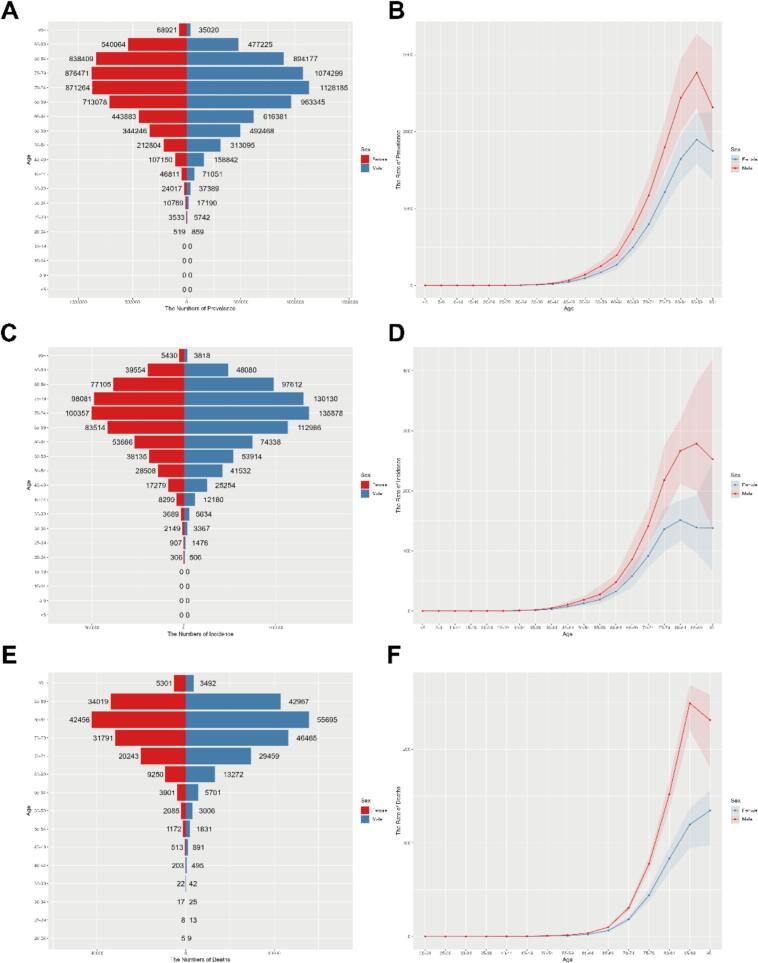
Age-specific numbers and age-standardized prevalence, incidence, and mortality rates of PD in GBD data, 2021. (A) Age-specifific prevalence number. (B) Age-standardized prevalence rate. (C) Age-specifific incidence number. (D) Age-standardized incidence rate. (E) Age-specifific mortality number. (F) Age-standardized mortality rate. PD, parkinson's disease.

Fig. 3 provides a clear picture of the global distribution of PD prevalence, incidence and mortality by age and sex in 2021. It is observed that the prevalence of PD is higher among people over 50 years of age, with a rapid increase in the number of patients between 60 and 89 years of age. Further analysis reveals that the peak in the number of cases occurs in the 80–89 age group for both males and females. A similar trend characterizes the incidence of the disease. The incidence of PD rises significantly from the age of 50, with the peak of the disease occurring between the ages of 65 and 84. Mortality rates for PD patients increase sharply after age 70, and peak in the 80–84 age range. The prevalence, incidence and mortality rates are significantly higher in males than in females in almost all age groups. Furthermore, age-standardized rates of DALYs, YLDs, and YLLs consistently demonstrated significantly higher values in males compared to females across all age groups, with particularly pronounced gender disparities emerging after age 65. All three metrics exhibited rapid acceleration in both sexes beyond this age threshold (Supplementary Fig. 1).

### 3.3 SDI burdens of PD

**Fig. 4:**
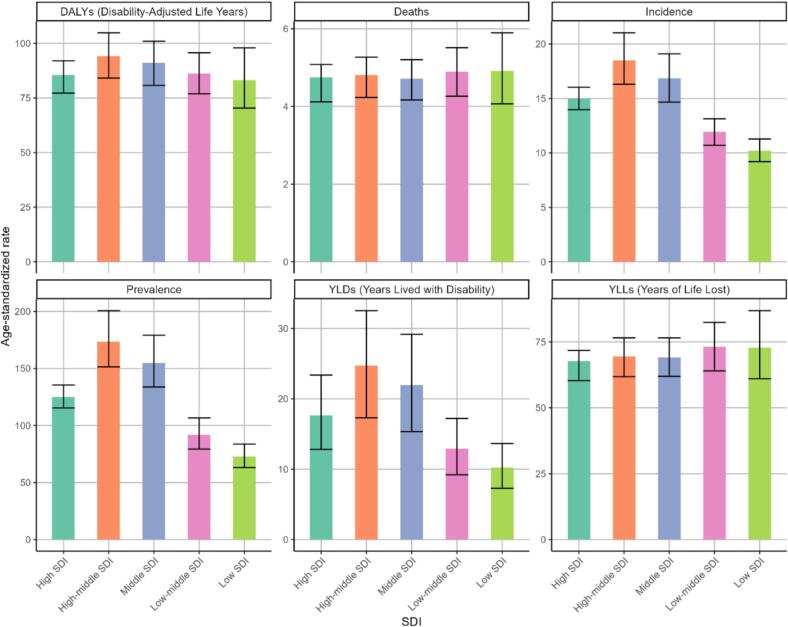
Burden of disease for PD in different SDI regions in 2021. PD, parkinson's disease; SDI, socio-demographic index.

After stratifying 204 countries or regions around the world according to the SDI, we observed that the burden of PD is heavier in regions with higher SDI levels (Fig. 4). Areas in the middle SDI, high-middle SDI, and high SDI tiers showed higher values of ASIR, ASPR, and ASDR for PD, whereas in low SDI and low-middle SDI areas, the corresponding ASIR, ASPR, and age-standardized YLDs for PD were lower. However, ASDR and ASMR showed minimal variation across SDI strata.

### 3.4 Trend analysis of PD over the period 1990 to 2021

**Fig. 5:**
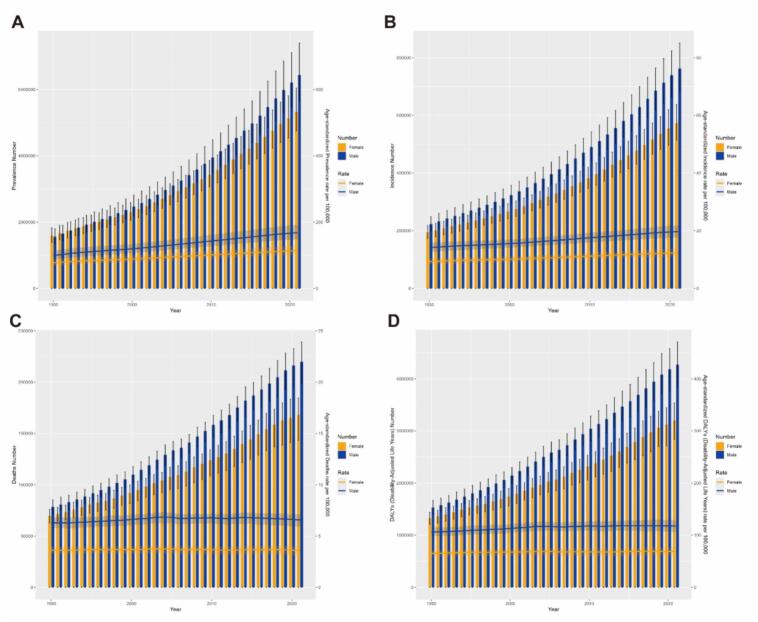
Trends in the all-age cases and age-standardized prevalence, incidence, mortality, and DALYs rates of PD by sex from 1990 to 2021. (A)Prevalence number and rate. (B)Incidence number and rate. (C) mortality number and rate. (D) DALYs number and rate. DALYs, disability-adjusted life years. DALYs, disability-adjusted life years; PD, parkinson's disease.

Analysis of Fig. 5 clearly shows that from 1990 to 2021, the number of PD cases, prevalence, incidence and DALYs in both the male and female populations have been increasing year by year—a trend reflected in the steady annual growth of global PD prevalence and incidence as shown by the total case numbers in Supplementary Fig. 2. Further observations revealed that ASPR and ASIR also climbed steadily over this 30-year span, with males experiencing significantly faster rates of increase than females. In contrast, however, ASMR and ASDR remained at relatively stable levels for both sexes during the same period. In addition, the number of YLDs and age-standardized YLDs for both males and females followed a yearly upward trend from 1990 to 2021. Although the number of YLDs increased annually over the past three decades, age-standardized YLDs did not fluctuate within a large range (Supplementary Fig. 3).

**Fig. 6:**
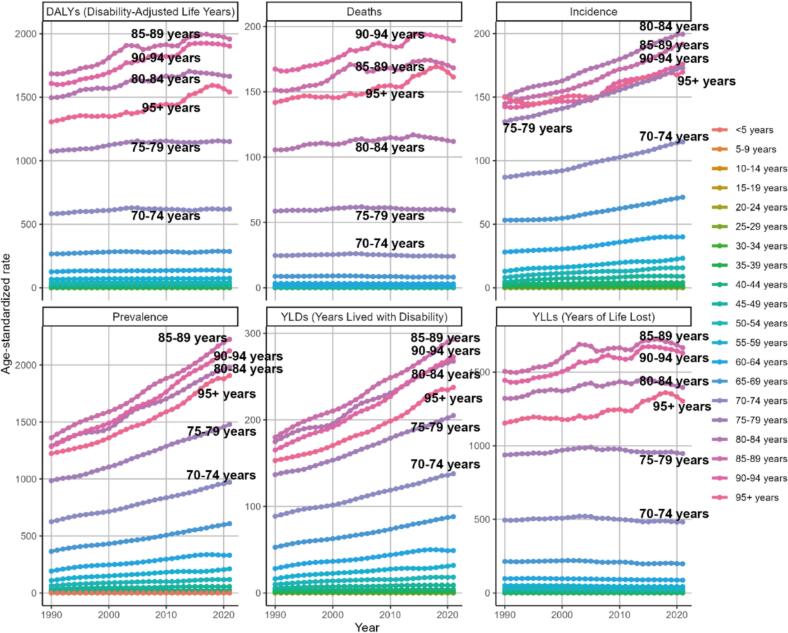
Trends in age-standardized rate in each of the indicators of PD by age group between 1990 and 2021. PD, Parkinson's disease.

Fig. 6 provides a clear picture of the evolution of PD by age from 1990 to 2021, with the help of six key age-standardized indicators (DALYs, deaths, incidence, prevalence, YLDs, and YLLs). Taken together, the disease burden associated with PD is significantly higher at relatively advanced ages, especially after age 70, and its increase is becoming more pronounced. However, age-standardized DALYs, mortality rates, and YLLs have shown a decreasing trend in the last 5 years, which has continued from year to year.

**Fig. 7:**
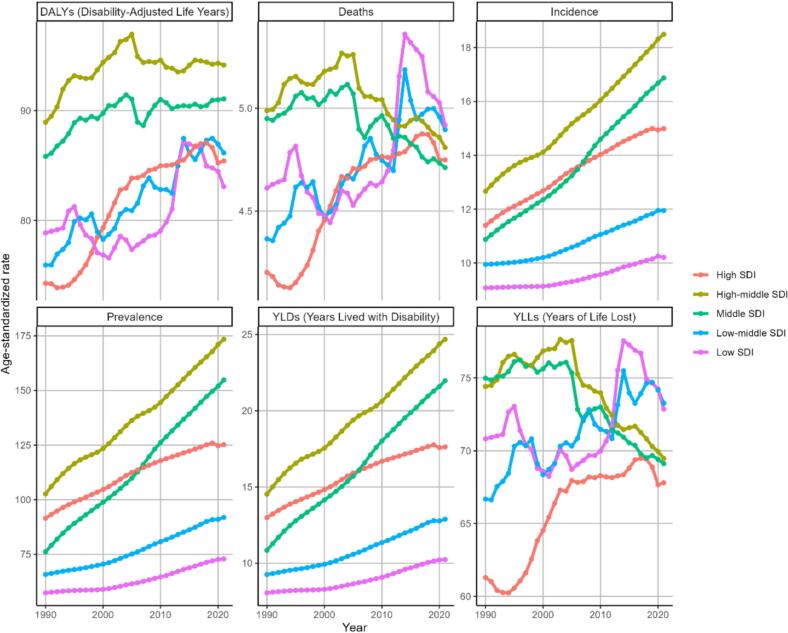
Trends in age-standardized rate in each of the indicators of PD by SDI regions between 1990 and 2021. PD, Parkinson's disease; SDI, socio-demographic index.

Fig. 7 provides a visual representation of how the six core age-standardized indicators of PD have changed between 1990 and 2021 in each of the regions based on the different SDI divisions. Looking at the overall picture, it can be seen that all SDI regions have shown an increase in the indicators over the last 30 years. Among them, the High-middle SDI and Middle SDI regions are particularly prominent, with ASDR ASIR, and age-standardized YLDs all showing increases. However, both ASMR and age-standardized YLLs in all SDI regions have shown a downward trend in the last 5 years.

### 3.5 EAPC

**Fig. 8:**
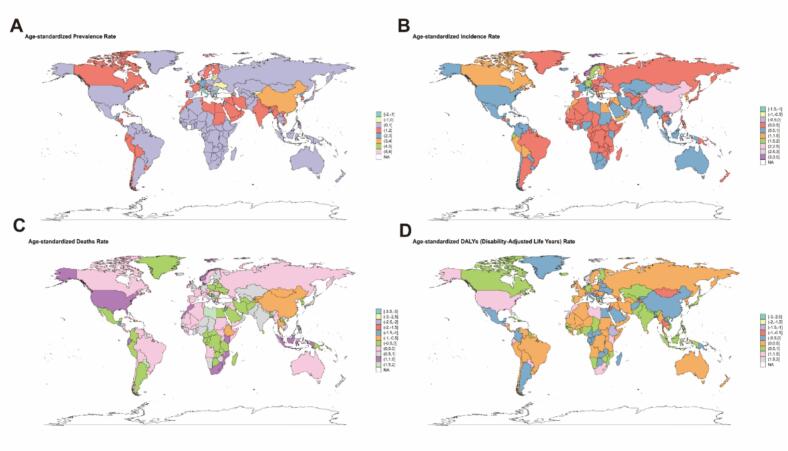
EAPC in the ASPR, ASIR, ASMR and ASDR from 1990 to 2021. (A) EAPC in the ASPR. (B) EAPC in the ASIR. (C) EAPC in the ASMR. (D) EAPC in the ASDR. EAPC, estimated annual percentage change; ASPR, age-standardized prevalence rate; ASIR, age-standardized incidence rate; ASMR, age-standardized mortality rate; ASDR, age-standardized DALY rate. DALYs, disability-adjusted life years; PD, parkinson's disease.

In all but a few of the more than 20 GBD regions around the world, the disease burden of PD has shown an increasing trend over time (Fig. 8 and Table 3). Specifically, in terms of ASPR and ASIR, Taiwan Province of China, Norway, and China consistently rank among the top three in terms of EAPC values. In terms of ASMR, Bhutan and Japan in Asia have higher numbers, with their EAPC values ranking first and second, respectively. In terms of ASDR, the top three regions in terms of EAPC value are all located in Asia, namely, Bhutan, Taiwan (Province of China), and Japan. The supplementary Fig. 4 details the dynamics of global PD prevalence, incidence, mortality and DALY numbers between 1990 and 2021. The burden of PD displays considerable variability across GBD regions. Hierarchical cluster analysis was conducted to identify regions with similar patterns of change in disease burden. According to Supplementary Fig. 5, which incorporated various pre-defined GBD analytical groupings, Andean Latin America (a GBD geographic region) and Asia exhibited a significant increase in both ASPR and age-standardized DALYs rate, whereas regions and groupings such as East Asia (GBD geographic region), the Basic Health System grouping, the World Bank Upper Middle Income grouping, and the World Bank's East Asia & Pacific region showed significant decreases.

**Table 3:** Top 10 countries and regions of EAPC.

Prevalence		Incidence		Deaths		DALYs	
Taiwan (Province of China)	5.9 (5.31–6.49)	Taiwan (Province of China)	4.54 (4.03–5.06)	Bhutan	2.66 (1.39–3.94)	Bhutan	2.24 (1.15–3.35)
Norway	5.26 (4.43–6.09)	China	3.65 (3.26–4.04)	Japan	2.62 (1.33–3.93)	Taiwan (Province of China)	2.21 (1.76–2.67)
China	4.61 (4.22–5)	Norway	3.43 (2.6–4.26)	Qatar	−2.54 (−5.65–0.66)	Japan	2.18 (0.99–3.39)
Republic of Korea	3.65 (3.16–4.14)	Republic of Korea	3.15 (2.66–3.64)	Republic of Korea	2.32 (1.68–2.96)	Republic of Korea	2.16 (1.62–2.71)
Democratic People's Republic of Korea	3.27 (2.97–3.56)	Germany	2.67 (1.67–3.67)	Albania	2.29 (1.68–2.9)	Libya	2.14 (1.06–3.23)
Germany	2.99 (1.96–4.04)	Ecuador	2.63 (2.04–3.23)	Timor-Leste	2.23 (0.54–3.95)	Honduras	2.09 (0.93–3.26)
Syrian Arab Republic	2.94 (1.62–4.27)	Bhutan	2.59 (1.63–3.56)	Lithuania	2.22 (1.37–3.08)	Ecuador	2.08 (1.37–2.8)
Ecuador	2.92 (2.33–3.52)	Syrian Arab Republic	2.45 (1.14–3.77)	Ecuador	2.21 (1.4–3.01)	Albania	2.06 (1.51–2.61)
Bhutan	2.84 (1.88–3.8)	Democratic People's Republic of Korea	2.39 (2.1–2.68)	Libya	2.19 (1.02–3.38)	Timor-Leste	2.01 (0.6–3.45)
Peru	2.75 (2.23–3.28)	United States Virgin Islands	2.35 (1.82–2.88)	Paraguay	2.18 (1.4–2.95)	Paraguay	1.99 (1.26–2.72)

EAPC, estimated annual percentage change. DALYs, disability-adjusted life years.

### 3.6 Future forecasts of global burden of PD

**Fig. 9:**
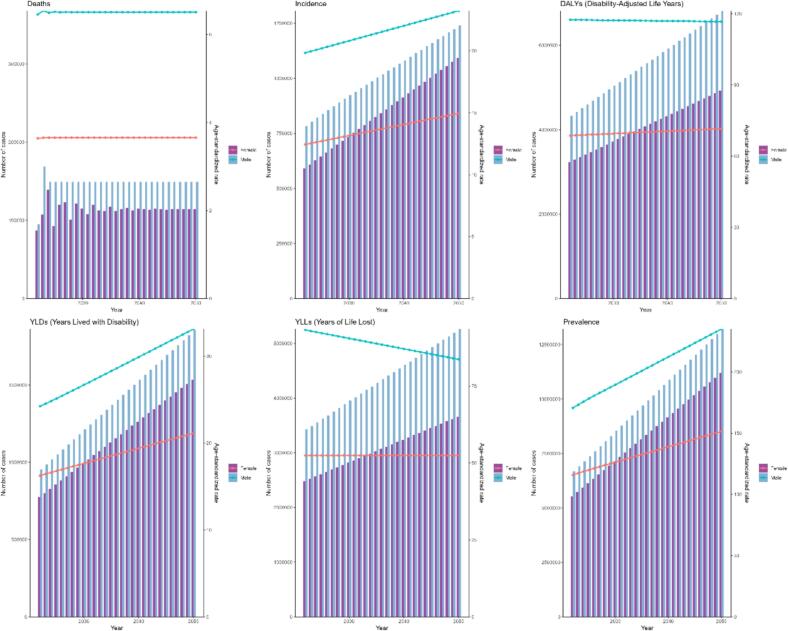
Future forecasts of PD global burden based on the ARIMA model. PD, Parkinson's disease; ARIMA, autoregressive integrated moving average model.

**Fig. 10:**
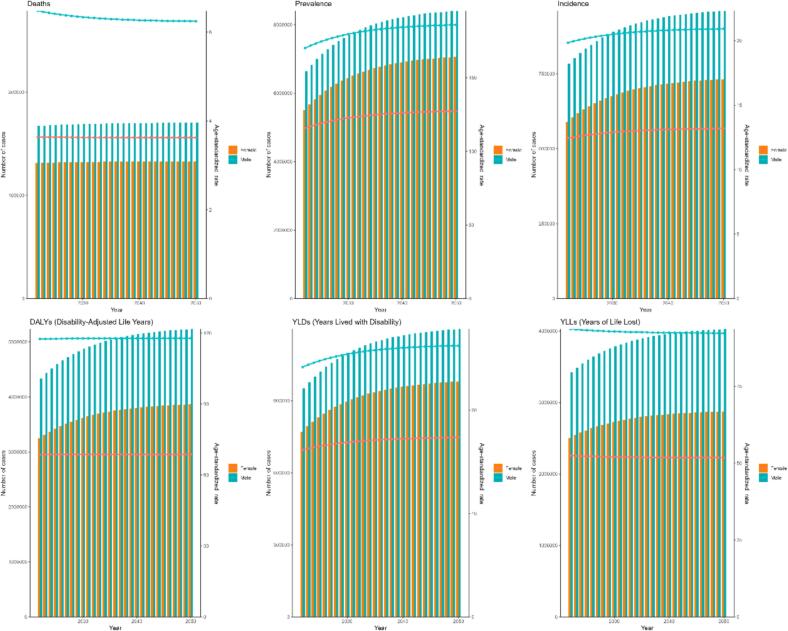
Future forecasts of PD global burden based on the ES model. PD, Parkinson's disease; ES, exponential smoothing.

To gain a clearer insight into the future trend of PD, we quantitatively projected the global burden of PD over the next 30 years by importing PD data stratified by sex from 1990 to 2021 into the ARIMA model. Further studies found that the global burden of PD is expected to change significantly between 2021 and 2050, and that the trends presented by different indicators vary (Fig. 9 and Table 4). Overall, the ASPR and ASIR will show a significant increase in both males and females over the next 30 years. Specific data show that the ASPR for males is expected to increase from about 168 cases per 100,000 people in 2021 to about 235 cases per 100,000 people in 2050, which means an increase of about 40% in this 30-year span; while the ASPR for females is expected to increase from about 114 cases per 100,000 people in 2021 to about 151 cases per 100,000 people in 2050, which is an increase of about 32 percent. However, although the prevalence and incidence of PD are expected to rise significantly over the next 30 years, the ASMR and ASDR for PD are not expected to change significantly. In terms of specific values, the ASDR for males will be nearly flat from about 6.6 cases per 100,000 people in 2021 to about 6.5 cases per 100,000 people in 2050; the ASMR for females will remain at about 3.5 cases per 100,000 people from about 3.6 cases per 100,000 people in 2021 to about 3.5 cases per 100,000 people in 2050, which indicates that the ASMR and ASDR for both males and females will remain stable without significant fluctuations in the next 30 years. In addition, we also used the ES model to quantify the global burden of PD in 2025, and the results were broadly similar to the trends predicted by the ARIMA model (Fig. 10 and Supplementary Table 1). Similarly, ASPR and ASIR will increase significantly over the next 30 years for both men and women, although at a slightly slower rate than in the ARIMA model. The ASMR and ASDR in PD predicted by the ES model are almost identical to those predicted by the ARIMA model.

**Table 4:** Future forecasts of PD global burden based on the ARIMA model (age-standardized prevalence, incidence, mortality and DALYs rates).

	Prevalence		Incidence		Mortality		DALYs	
Year	male	female	male	female	male	female	male	female
2022	170.65	115.91	19.84	12.45	6.46	3.64	117.44	68.67
2023	173.19	117.29	19.96	12.54	6.54	3.65	117.41	68.77
2024	175.72	118.63	20.08	12.63	6.50	3.66	117.39	68.87
2025	178.17	119.94	20.20	12.71	6.52	3.66	117.36	68.98
2026	180.53	121.23	20.33	12.80	6.51	3.66	117.33	69.08
2027	182.81	122.51	20.45	12.89	6.51	3.66	117.30	69.18
2028	185.06	123.78	20.57	12.98	6.51	3.66	117.27	69.29
2029	187.29	125.05	20.69	13.07	6.51	3.66	117.25	69.39
2030	189.53	126.31	20.81	13.16	6.51	3.66	117.22	69.49
2031	191.78	127.57	20.93	13.25	6.51	3.66	117.19	69.59
2032	194.05	128.82	21.05	13.34	6.51	3.66	117.16	69.70
2033	196.32	130.08	21.18	13.43	6.51	3.66	117.14	69.80
2034	198.60	131.34	21.30	13.52	6.51	3.66	117.11	69.90
2035	200.88	132.59	21.42	13.61	6.51	3.66	117.08	70.01
2036	203.16	133.84	21.54	13.70	6.51	3.66	117.05	70.11
2037	205.43	135.10	21.66	13.79	6.51	3.66	117.03	70.21
2038	207.71	136.35	21.78	13.88	6.51	3.66	117.00	70.32
2039	209.98	137.61	21.90	13.96	6.51	3.66	116.97	70.42
2040	212.25	138.86	22.03	14.05	6.51	3.66	116.94	70.52
2041	214.53	140.11	22.15	14.14	6.51	3.66	116.92	70.62
2042	216.80	141.37	22.27	14.23	6.51	3.66	116.89	70.73
2043	219.07	142.62	22.39	14.32	6.51	3.66	116.86	70.83
2044	221.35	143.88	22.51	14.41	6.51	3.66	116.83	70.93
2045	223.62	145.13	22.63	14.50	6.51	3.66	116.80	71.04
2046	225.89	146.38	22.75	14.59	6.51	3.66	116.78	71.14
2047	228.17	147.64	22.88	14.68	6.51	3.66	116.75	71.24
2048	230.44	148.89	23.00	14.77	6.51	3.66	116.72	71.35
2049	232.72	150.14	23.12	14.86	6.51	3.66	116.69	71.45
2050	234.99	151.40	23.24	14.95	6.51	3.66	116.67	71.55

ARIMA, autoregressive Integrated Moving Average model; DALYs, disability-adjusted life years.

## 4 Discussion

This study confirms the substantial and persistent global burden of Parkinson's disease. PD poses a dual challenge, impairing health while exerting a heavy and inequitable socioeconomic impact. The economic burden disparities are stark: high-SDI countries and regions face high per-capita costs, while in low- and middle-SDI countries and regions, care costs consume a disproportionate share of household income and GDP, heightening the risk of catastrophic health expenditure [16], [17], [18], [19]. This disparity underscores PD's role as a significant driver of health inequality, placing immense strain on healthcare systems and household finances globally[19], [20].

Significant heterogeneity in PD burden exists across global regions, with China and Japan in Asia alongside Canada in North America demonstrating markedly higher ASPR and ASIR values, whereas most African nations exhibited comparatively lower burdens. This observed geographical disparity may be attributed to multifactorial determinants including environmental exposures, lifestyle patterns, healthcare resource disparities, and differential population aging trajectories [21].Multiple studies support an association between exposure to specific environmental toxins and an increased risk of PD. Exposure to pesticides widely used in agricultural regions (e.g., rotenone, paraquat) and industrial heavy metals (e.g., manganese, lead) has been established by epidemiological and mechanistic research as potential risk factors for PD [22], [23], [24]. Differences in industrialization levels, agricultural practices, and environmental regulatory policies across countries and regions may lead to varying population exposure levels, thereby influencing the PD burden [25]. Lifestyle variations may also impact PD risk. For instance, cigarette smoking and caffeine intake are associated with a lower PD risk (although smoking itself poses significant health hazards), while certain dietary habits or physical activity levels may exert modulatory effects [26], [27]. These lifestyle factors exhibit significant variation among populations with diverse cultural and socioeconomic backgrounds globally.

Disparities in healthcare resource accessibility and diagnostic capacity significantly impact the identification and reporting of PD cases. High SDI regions typically possess more robust healthcare infrastructure, advanced diagnostic technologies (e.g., dopamine transporter imaging using single-photon emission computed tomography, DAT-SPECT), and a larger cadre of specialized neurologists. This often leads to higher case detection rates and more accurate burden assessments [8], [16]. Conversely, in low-SDI regions, limited healthcare resources and lower disease awareness may result in substantial underdiagnosis and under-ascertainment of PD cases within statistical systems, leading to underestimation of the disease burden [8].

Furthermore, variations in treatment accessibility influence disease progression, complication rates, and ultimate health outcomes (e.g., mortality, disability rates). The primary non-modifiable risk factor for PD is advanced age [28], [29]. Consequently, the degree of population aging is a key driver of PD burden. Many high-burden countries and regions (e.g., Japan, China, Canada) are undergoing rapid and profound demographic transitions, characterized by a significantly increasing proportion of elderly individuals [10], [11]. For instance, Japan possesses the highest global aging rate, which is closely associated with its elevated PD burden. In contrast, several low-burden African nations maintain relatively younger population structures.

Therefore, the heterogeneous global pattern of PD burden results from the combined effects of the aforementioned environmental, lifestyle, healthcare system, and sociodemographic factors—particularly aging [10]. Understanding the relative contribution of these drivers is crucial for formulating region-specific, effective prevention and control strategies.

This study identified significantly higher prevalence, incidence, and mortality rates among males compared to females across nearly all age groups. This sex-based disparity likely stems from multifactorial interactions involving complex biological and environmental determinants. Males may exhibit heightened susceptibility to specific environmental neurotoxins (e.g., pesticides, heavy metals) and display variations in sex-linked genetic predisposition patterns [21], [30]. Mounting evidence indicates that sex hormones, particularly estrogen, confer neuroprotective properties on dopaminergic neurons. This biological mechanism likely contributes to the attenuated susceptibility to Parkinson's disease pathogenesis observed in females during their estrogen-proficient premenopausal phase [30], [31], [32], [33], [34]. This study also confirms other studies showing that PD is strongly associated with advancing age[28], [29] with peak incidence between the ages of 65 and 84. These pronounced disparities underscore the imperative for integrated consideration of sex-specific and age-stratified determinants in both elucidating Parkinson's disease pathogenesis and formulating targeted prevention/management strategies.

In terms of SDI, ASIR, ASPR and age-standardized YLDs are higher for PD in middle SDI, high-middle SDI and high SDI areas. This may be related to environmental pollution, lifestyle changes, and more advanced diagnostic techniques[35], [36], [37]. Although the overall burden of Parkinson's disease is rising more sharply in high-SDI regions, all regions have recently enhanced their focus on PD treatment and management. Through measures such as increased medical investment, improved technology, and public health education, premature mortality and years of life lost appear stable, positively impacting patient survival and quality of life [38], [39], [40]. In contrast, low and low-middle SDI regions showed only modest increases in PD burden, potentially due to limited medical resources, underdiagnosis, and younger population structures[8]. Despite a lower overall burden, these regions exhibited a higher ASMR, which increased sharply after 2010 and surpassed that of high-SDI regions after 2012, likely a consequence of weaker socioeconomic and healthcare conditions leading to worse patient outcomes. This disparity underscores the need for stratified global strategies: high-SDI regions should focus on optimizing disease management and quality of life, while low-SDI regions require fundamental improvements in healthcare infrastructure and support.

Rapidly increasing EAPCs in PD incidence, prevalence, and DALYs in selected Asian regions (including Taiwan Province of China, Bhutan, and Japan) highlight a sharply growing disease burden [41]. This trend, strongly associated with population aging, necessitates age-specific public health interventions. Asian nations should enhance collaboration through sharing protocols and coordinating research resources, while global stakeholders must develop tailored strategies based on regional profiles to improve outcomes and patient quality of life across diverse healthcare settings.

When interpreting the observed increases in PD burden, the influence of evolving methodologies must be considered. Advances in diagnostic criteria (e.g., the adoption of MDS criteria) and the growing use of neuroimaging (e.g., DAT-SPECT) have improved the detection of early-stage and atypical cases, thereby contributing to the reported rises in incidence and prevalence [38], [39], [40]. Concurrently, enhanced disease awareness and the establishment of national registries have improved case inclusion in statistical systems [42]. The magnitude of this effect, however, varies regionally. Sophisticated healthcare systems in high-SDI regions may more comprehensively capture genuine burden increases, whereas low-SDI regions likely suffer from substantial under-ascertainment due to limited resources, leading to potential underestimation [8], [43]. Therefore, the global upward trend, particularly the disparities across SDI tiers, likely reflects a combination of true shifts in disease burden and improvements in diagnostic capabilities and reporting completeness. The GBD study employs standardized modeling approaches (e.g., DisMod-MR, a Bayesian *meta*-regression tool) to integrate heterogeneous data sources of varying quality, utilizing covariates (e.g., Healthcare Access and Quality Index) for adjustment. This methodology aims to minimize such biases and provide comparable estimates across time and geography [6], [13]. Nevertheless, cautious interpretation of long-term secular trends and cross-regional differences remains imperative, explicitly accounting for the influence of these methodological factors.

Forecasting models indicate substantial global increases in PD prevalence and incidence over the next three decades, with a notably faster rise in males, while mortality rates remain stable. This trend necessitates a dual strategy: enhancing early screening for high-risk populations (especially elderly males), and sustaining advancements in therapy and disease management to improve patient quality of life. Aligning healthcare resources with these projections is crucial for mitigating the escalating disease burden.

## 5 Limitation

This study provides comprehensive data and analysis for understanding the global disease burden of PD, yet several limitations warrant consideration. For instance, GBD datasets may contain inherent uncertainties and potential biases, necessitating optimization of data collection and analytical methodologies in future research. Additionally, the observed lag in global infrastructure data must be accounted for, as more real-world studies are essential to validate findings and enable more accurate, holistic assessments. In terms of forecasting, ARIMA and ES models make predictions under the assumption that historical trends will continue, which poses inherent risks for long-term predictions. The burden of PD is influenced by numerous dynamic underlying factors (e.g., breakthroughs in diagnostic/therapeutic technologies, dramatic shifts in environmental exposures/lifestyle behaviors, accelerated population aging, major disruptive events) [44]. These models are inherently unable to anticipate such structural shifts, potentially leading to forecast bias. The accuracy of the predictive outputs is highly dependent on the quality and completeness of the GBD historical input data. Although GBD data are considered authoritative, the estimates themselves carry inherent uncertainty – particularly in data-scarce regions. This uncertainty propagates into the forecast results.

## 6 Conclusion

Our analysis of the GBD 2021 data confirms that Parkinson's disease remains a substantial and growing global public health challenge. The disease burden demonstrates clear sex (male predominance) and age (older populations) associations, alongside significant geographical heterogeneity. While higher SDI regions report greater incidence, lower-SDI regions may experience more severe health outcomes. Projections for the next three decades indicate a continued rise in global prevalence and incidence, particularly among males. Collectively, these findings provide critical evidence for informing targeted prevention strategies and optimizing healthcare resource allocation across different regions.

## Data available statement

All research data in this work are obtained from the GBD 2021 (https://ghdx.healthdata.org/gbd-results-tool).

## CRediT authorship contribution statement

**Sen Wang:** Writing – original draft, Data curation. **Yujie Che:** Conceptualization. **Yiqun Lin:** Writing – original draft, Formal analysis, Data curation. **Yukang Zhang:** Methodology. **Wen He:** Writing – review & editing, Validation, Supervision. **Wei Zhang:** Writing – review & editing, Visualization, Validation, Supervision, Resources.

## Funding

This study has received the funding by grants from the National Nature Science Foundation of China (8200184, 82271995).

## Declaration of competing interest

The authors declare that they have no known competing financial interests or personal relationships that could have appeared to influence the work reported in this paper.

## References

[1] Morris H.R., Spillantini M.G., Sue C.M., Williams-Gray C.H. (2024). The pathogenesis of Parkinson's disease. Lancet.

[2] Leite Silva A.B.R., Goncalves de Oliveira R.W., Diogenes G.P., de Castro Aguiar M.F., Sallem C.C., Lima M.P.P., de Albuquerque Filho L.B., Peixoto de Medeiros S.D., Penido de Mendonca L.L., de Santiago Filho P.C., Nones D.P., da Silva Cardoso P.M.M., Ribas M.Z., Galvao S.L., Gomes G.F., Bezerra de Menezes A.R., Dos Santos N.L., Mororo V.M., Duarte F.S., Dos Santos J.C.C. (2023). Premotor, nonmotor and motor symptoms of Parkinson's disease: a new clinical state of the art. Ageing Res. Rev..

[3] Diseases G.B.D., Injuries C. (2020). Global burden of 369 diseases and injuries in 204 countries and territories, 1990-2019: a systematic analysis for the Global Burden of Disease Study 2019. Lancet.

[4] Cen J., Wang Q., Cheng L., Gao Q., Wang H., Sun F. (2024). Global, regional, and national burden and trends of migraine among women of childbearing age from 1990 to 2021: insights from the Global Burden of Disease Study 2021. J. Headache Pain.

[5] Feigin V.L., Vos T., Nichols E., Owolabi M.O., Carroll W.M., Dichgans M., Deuschl G., Parmar P., Brainin M., Murray C. (2020). The global burden of neurological disorders: translating evidence into policy. Lancet Neurol..

[6] Diseases G.B.D., Injuries C. (2024). Global incidence, prevalence, years lived with disability (YLDs), disability-adjusted life-years (DALYs), and healthy life expectancy (HALE) for 371 diseases and injuries in 204 countries and territories and 811 subnational locations, 1990-2021: a systematic analysis for the Global Burden of Disease Study 2021. Lancet.

[7] Darweesh S.K., Koudstaal P.J., Stricker B.H., Hofman A., Ikram M.A. (2016). Trends in the incidence of Parkinson disease in the general population: the rotterdam study. Am. J. Epidemiol..

[8] G.B.D.N. Africa, C. the Middle East Neurology, The burden of neurological conditions in north Africa and the Middle East, 1990-2019: a systematic analysis of the Global Burden of Disease Study 2019, Lancet Glob Health 12(6) (2024) e960-e982. 10.1016/S2214-109X(24)00093-7 PMC11099299 38604203

[9] Wang S., Jiang Y., Yang A., Meng F., Zhang J. (2024). The expanding burden of neurodegenerative diseases: an unmet medical and social need. Aging Dis..

[10] Yang Q., Chang X., Li S., Li X., Kang C., Yuan W., Lv G. (1990). Disease burden of Parkinson's disease in Asia and its 34 countries and territories from 1990 to 2021: findings from the global burden of disease study 2021. Neuroepidemiology.

[11] Xu T., Dong W., Liu J., Yin P., Wang Z., Zhang L., Zhou M. (2024). Disease burden of Parkinson's disease in China and its provinces from 1990 to 2021: findings from the global burden of disease study 2021. Lancet Reg. Health West Pac..

[12] Yang X., Zhang T., Zhang Y., Chen H., Sang S. (1990). Global burden of COPD attributable to ambient PM2.5 in 204 countries and territories, 1990 to 2019: a systematic analysis for the global burden of disease study 2019. Sci. Total Environ..

[13] Sang S., Chu C., Zhang T., Chen H., Yang X. (2022). The global burden of disease attributable to ambient fine particulate matter in 204 countries and territories, 1990-2019: a systematic analysis of the Global Burden of Disease Study 2019. Ecotoxicol. Environ. Saf..

[14] Foreman K.J., Lozano R., Lopez A.D., Murray C.J. (2012). Modeling causes of death: an integrated approach using CODEm. Popul. Health Metr..

[15] Stevens G.A., Alkema L., Black R.E., Boerma J.T., Collins G.S., Ezzati M., Grove J.T., Hogan D.R., Hogan M.C., Horton R., Lawn J.E., Marusic A., Mathers C.D., Murray C.J., Rudan I., Salomon J.A., Simpson P.J., Vos T., Welch V. (2016). Guidelines for accurate and transparent health estimates reporting: the GATHER statement. Lancet.

[16] Kowal S.L., Dall T.M., Chakrabarti R., Storm M.V., Jain A. (2013). The current and projected economic burden of Parkinson's disease in the United States. Mov. Disord..

[17] Yang W., Hamilton J.L., Kopil C., Beck J.C., Tanner C.M., Albin R.L., Ray Dorsey E., Dahodwala N., Cintina I., Hogan P., Thompson T. (2020). Current and projected future economic burden of Parkinson's disease in the U.S. NPJ Parkinsons Dis..

[18] Dotchin C.L., Msuya O., Walker R.W. (2007). The challenge of Parkinson's disease management in Africa. Age Ageing.

[19] Prado M., Jamora R.D. (2020). Cost of Parkinson's disease among Filipino patients seen at a public tertiary hospital in Metro Manila. J. Clin. Neurosci..

[20] Schrag A., Hovris A., Morley D., Quinn N., Jahanshahi M. (2006). Caregiver-burden in Parkinson's disease is closely associated with psychiatric symptoms, falls, and disability. Parkinsonism Relat. Disord..

[21] Ben-Shlomo Y., Darweesh S., Llibre-Guerra J., Marras C., San Luciano M., Tanner C. (2024). The epidemiology of Parkinson's disease. Lancet.

[22] Tanner C.M., Kamel F., Ross G.W., Hoppin J.A., Goldman S.M., Korell M., Marras C., Bhudhikanok G.S., Kasten M., Chade A.R., Comyns K., Richards M.B., Meng C., Priestley B., Fernandez H.H., Cambi F., Umbach D.M., Blair A., Sandler D.P., Langston J.W. (2011). Rotenone, paraquat, and Parkinson's disease. Environ. Health Perspect..

[23] Gunnarsson L.G., Bodin L. (2019). Occupational exposures and neurodegenerative diseases-a systematic literature review and meta-analyses. Int. J. Environ. Res. Public Health.

[24] Weisskopf M.G., Weuve J., Nie H., Saint-Hilaire M.H., Sudarsky L., Simon D.K., Hersh B., Schwartz J., Wright R.O., Hu H. (2010). Association of cumulative lead exposure with Parkinson's disease. Environ. Health Perspect..

[25] Bellou V., Belbasis L., Tzoulaki I., Evangelou E., Ioannidis J.P. (2016). Environmental risk factors and Parkinson's disease: an umbrella review of meta-analyses. Parkinsonism Relat. Disord..

[26] Ascherio A., Schwarzschild M.A. (2016). The epidemiology of Parkinson's disease: risk factors and prevention. Lancet Neurol..

[27] Palacios N., Gao X., McCullough M.L., Jacobs E.J., Patel A.V., Mayo T., Schwarzschild M.A., Ascherio A. (2011). Obesity, diabetes, and risk of Parkinson's disease. Mov. Disord..

[28] Collier T.J., Kanaan N.M., Kordower J.H. (2017). Aging and Parkinson's disease: different sides of the same coin?. Mov. Disord..

[29] Vila M. (2019). Neuromelanin, aging, and neuronal vulnerability in Parkinson's disease. Mov. Disord..

[30] Cerri S., Mus L., Blandini F. (2019). Parkinson's disease in women and men: what's the difference?. J. Parkinsons Dis..

[31] Gillies G.E., Pienaar I.S., Vohra S., Qamhawi Z. (2014). Sex differences in Parkinson's disease. Front. Neuroendocrinol..

[32] Jurado-Coronel J.C., Cabezas R., Avila Rodriguez M.F., Echeverria V., Garcia-Segura L.M., Barreto G.E. (2018). Sex differences in Parkinson's disease: features on clinical symptoms, treatment outcome, sexual hormones and genetics. Front. Neuroendocrinol..

[33] Georgiev D., Hamberg K., Hariz M., Forsgren L., Hariz G.M. (2017). Gender differences in Parkinson's disease: a clinical perspective. Acta Neurol. Scand..

[34] Moisan F., Kab S., Mohamed F., Canonico M., Le Guern M., Quintin C., Carcaillon L., Nicolau J., Duport N., Singh-Manoux A., Boussac-Zarebska M., Elbaz A. (2016). Parkinson disease male-to-female ratios increase with age: French nationwide study and meta-analysis. J. Neurol. Neurosurg. Psychiatry.

[35] Dorsey E.R., Sherer T., Okun M.S., Bloem B.R. (2018). The emerging evidence of the Parkinson pandemic. J. Parkinsons Dis..

[36] Bergstra A.D., Been J.V., Burdorf A. (2022). The association of specific industry-related air pollution with occurrence of chronic diseases: a register-based study. Environ. Res..

[37] Owolabi J.O. (2021). Neuroepidemiology: perspectives from Africa. Neuroepidemiology.

[38] Schrag A., Ben-Shlomo Y., Quinn N. (2002). How valid is the clinical diagnosis of Parkinson's disease in the community?. J. Neurol. Neurosurg. Psychiatry.

[39] Postuma R.B., Berg D., Stern M., Poewe W., Olanow C.W., Oertel W., Obeso J., Marek K., Litvan I., Lang A.E., Halliday G., Goetz C.G., Gasser T., Dubois B., Chan P., Bloem B.R., Adler C.H., Deuschl G. (2015). MDS clinical diagnostic criteria for Parkinson's disease. Mov. Disord..

[40] Hoglinger G.U., Adler C.H., Berg D., Klein C., Outeiro T.F., Poewe W., Postuma R., Stoessl A.J., Lang A.E. (2024). A biological classification of Parkinson's disease: the SynNeurGe research diagnostic criteria. Lancet Neurol..

[41] Zhong Q.Q., Zhu F. (2022). Trends in prevalence cases and disability-adjusted life-years of Parkinson's disease: findings from the global burden of disease study 2019. Neuroepidemiology.

[42] Rizzo G., Copetti M., Arcuti S., Martino D., Fontana A., Logroscino G. (2016). Accuracy of clinical diagnosis of Parkinson disease: a systematic review and meta-analysis. Neurology.

[43] G.B.D.P.s.D. Collaborators, Global, regional, and national burden of Parkinson's disease, 1990-2016: a systematic analysis for the Global Burden of Disease Study 2016, Lancet Neurol 17(11) (2018) 939-953. 10.1016/S1474-4422(18)30295-3 PMC6191528 30287051

[44] Bronskill S.E., Maclagan L.C., Maxwell C.J., Iaboni A., Jaakkimainen R.L., Marras C., Wang X., Guan J., Harris D.A., Emdin A., Jones A., Sourial N., Godard-Sebillotte C., Vedel I., Austin P.C., Swartz R.H. (2022). Trends in health service use for Canadian adults with Dementia and Parkinson disease during the first wave of the COVID-19 pandemic. JAMA Health Forum..

