# Supplementary figures and images for: Epidemiology of Parkinson’s disease – Global burden of disease research from 1990 to 2021 and future trend predictions
Source: Clin Park Relat Disord. 2026 Jan 9;14:100421. doi: 10.1016/j.prdoa.2026.100421 (PMC12828836; doi:10.1016/j.prdoa.2026.100421)

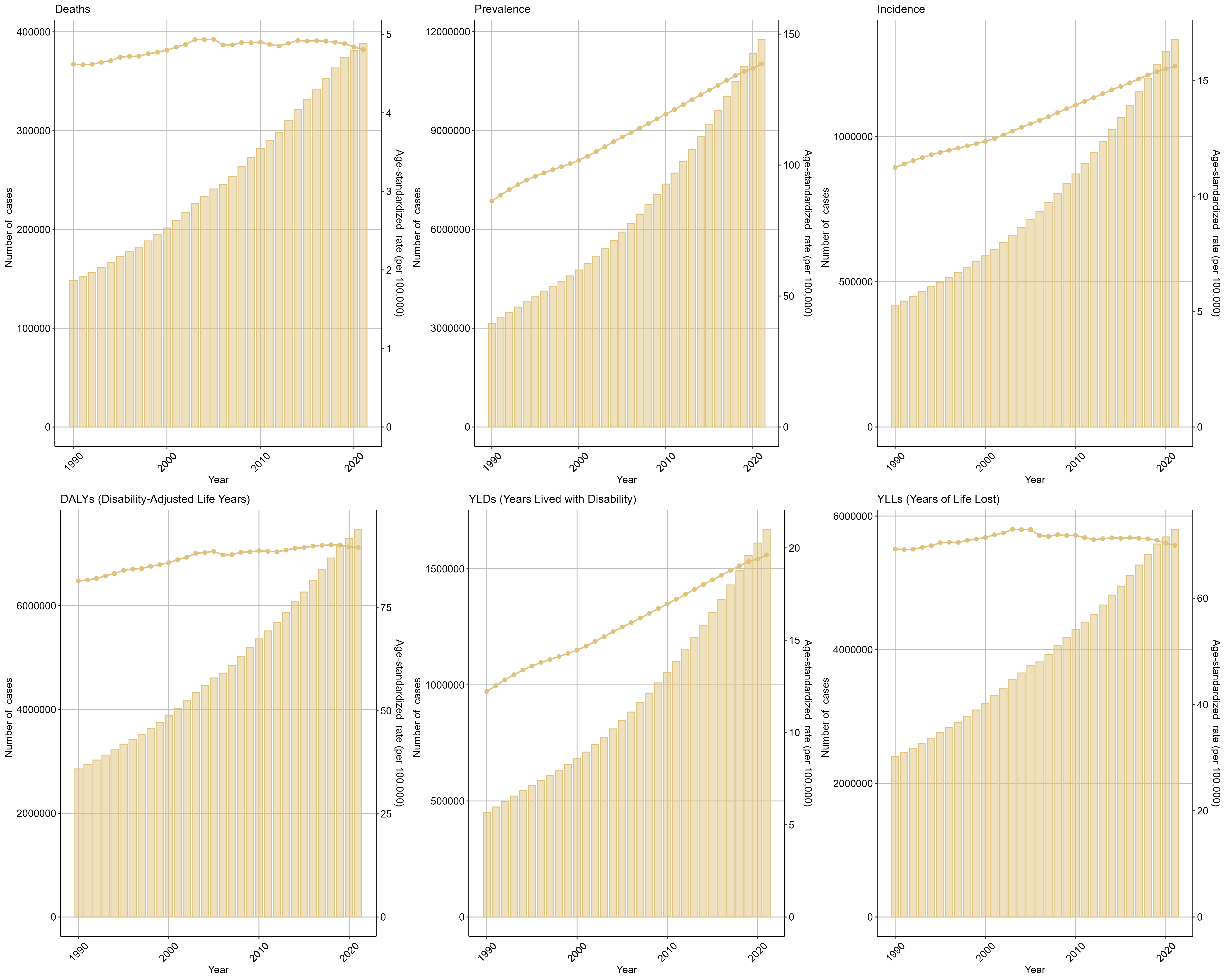

Supplement: Supplementary Data 2 [file mmc2.zip › Supplementary Figure 2.png]

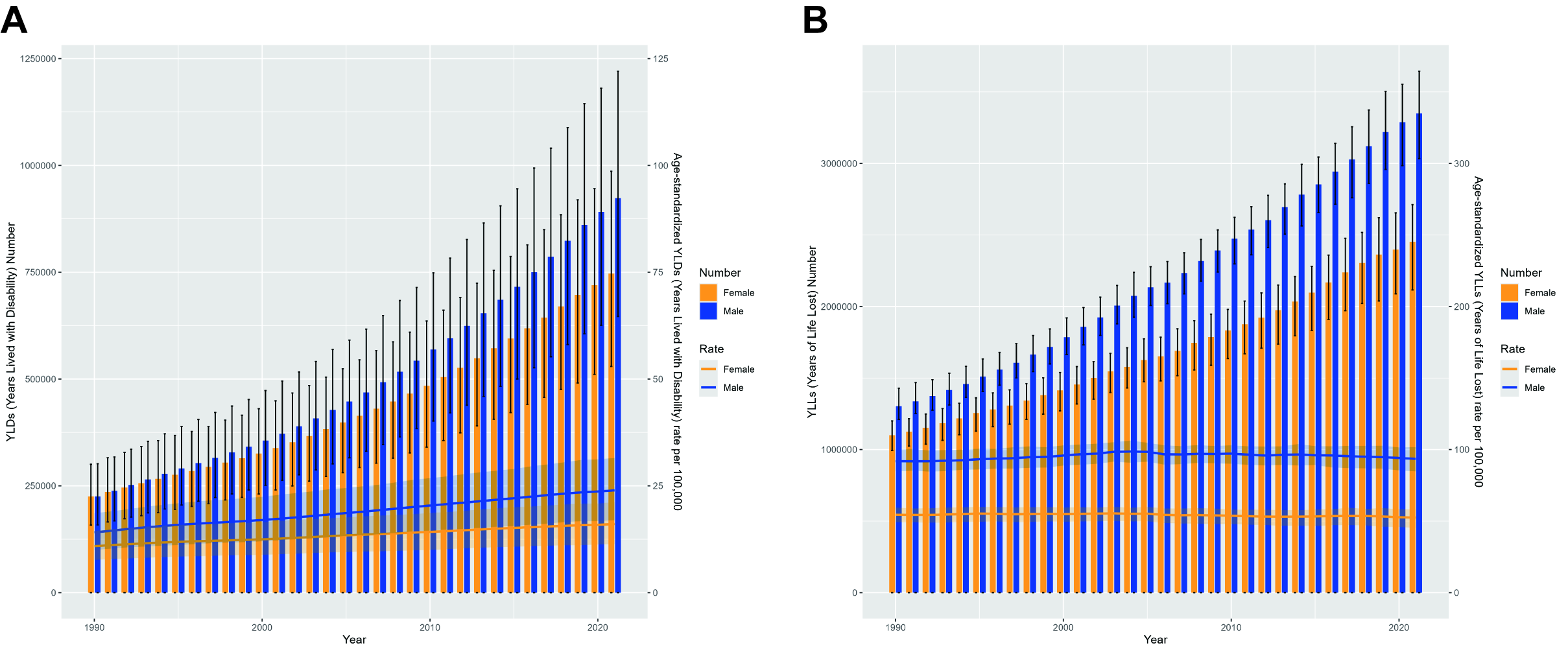

Supplement: Supplementary Data 3 [file mmc3.zip › Supplementary Figure 2.tif]

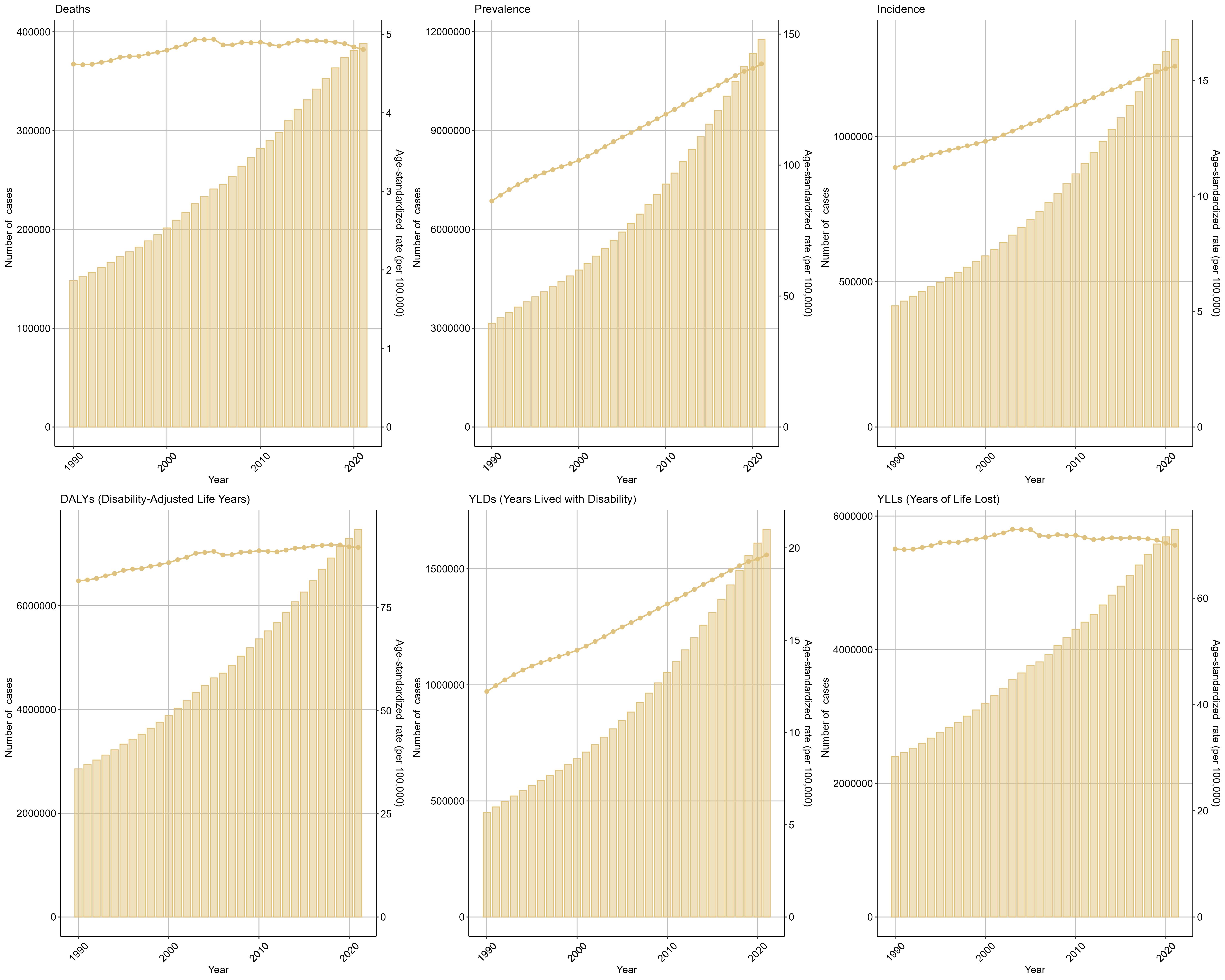

Supplement: Supplementary Data 4 [file mmc4.zip › Supplementary Figure 3.jpg]

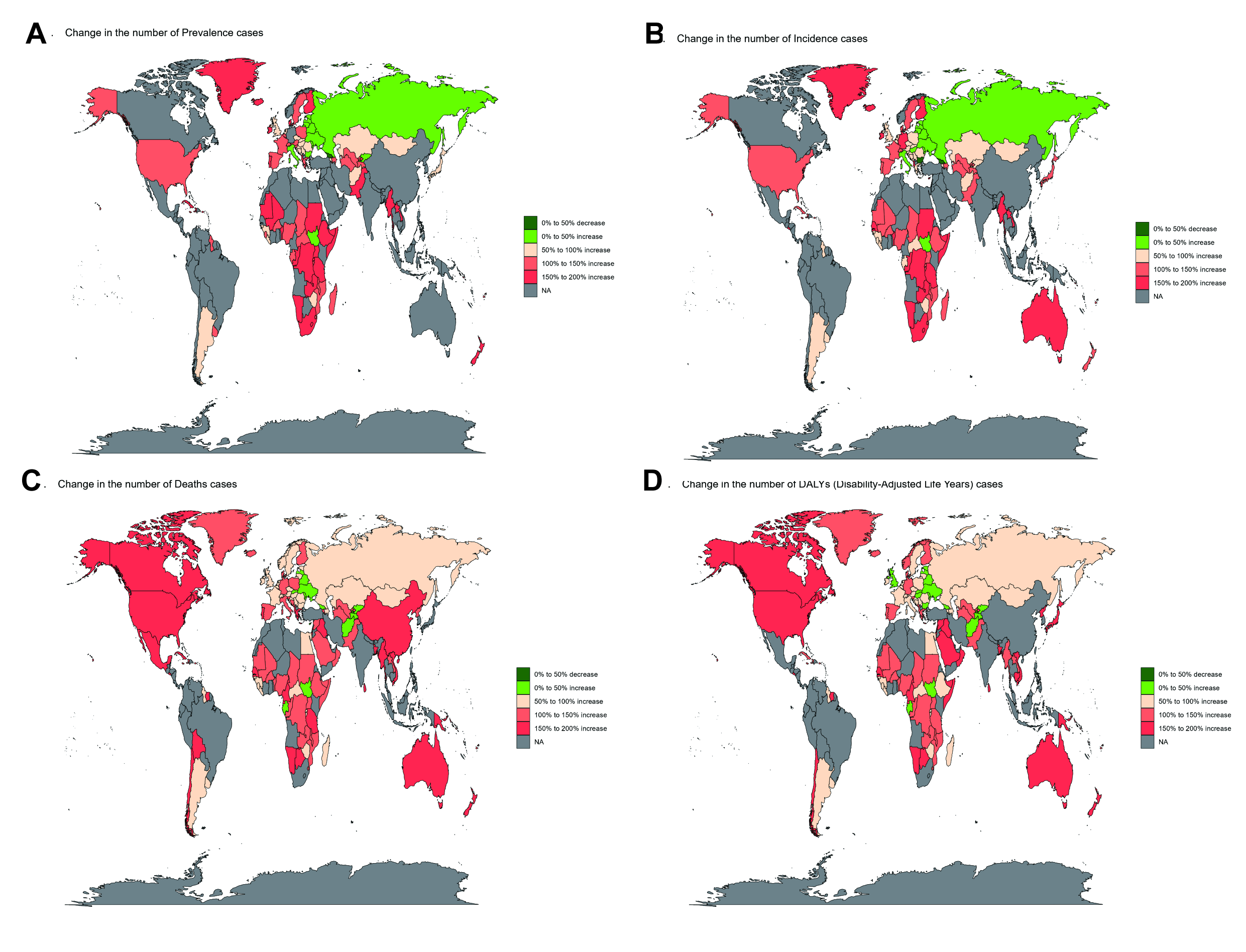

Supplement: Supplementary Data 6 [file mmc6.zip › Supplementary Figure4.tif]

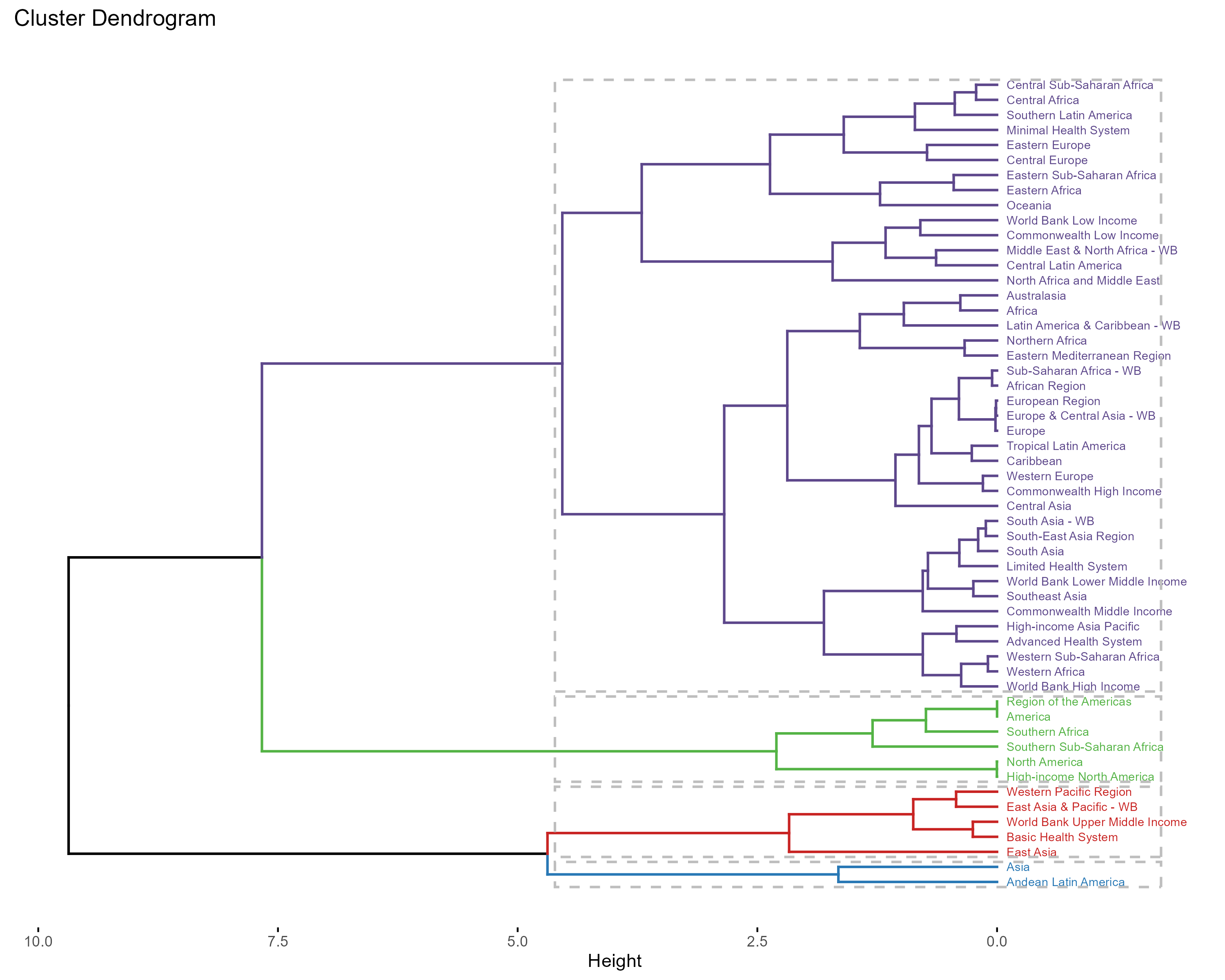

Supplement: Supplementary Data 7 [file mmc7.zip › Supplementary Figure5.png]
