# Supplementary material for: Epidemiology of Parkinson’s disease – Global burden of disease research from 1990 to 2021 and future trend predictions
Source: Clin Park Relat Disord. 2026 Jan 9;14:100421. doi: 10.1016/j.prdoa.2026.100421 (PMC12828836; doi:10.1016/j.prdoa.2026.100421)
Supplement: Supplementary Data 8 [file mmc8.doc]

**Supplementary Table1** Future forecasts of PD global burden based on the ES model(age-standardized prevalence, incidence, deaths and DALYs rates).

|  | Prevalence | | Incidence | | Deaths | | DALYs | |
| --- | --- | --- | --- | --- | --- | --- | --- | --- |
|  | male | female | male | female | male | female | male | female |
| 2022 | 170.09 | 115.80 | 19.85 | 12.44 | 6.48 | 3.64 | 117.51 | 68.58 |
| 2023 | 171.76 | 117.01 | 19.96 | 12.52 | 6.46 | 3.64 | 117.54 | 68.59 |
| 2024 | 173.26 | 118.11 | 20.06 | 12.59 | 6.43 | 3.63 | 117.58 | 68.60 |
| 2025 | 174.61 | 119.09 | 20.15 | 12.66 | 6.41 | 3.63 | 117.61 | 68.61 |
| 2026 | 175.83 | 119.98 | 20.24 | 12.72 | 6.40 | 3.63 | 117.63 | 68.62 |
| 2027 | 176.92 | 120.78 | 20.31 | 12.77 | 6.38 | 3.63 | 117.66 | 68.62 |
| 2028 | 177.91 | 121.49 | 20.38 | 12.81 | 6.36 | 3.63 | 117.68 | 68.63 |
| 2029 | 178.79 | 122.14 | 20.44 | 12.86 | 6.35 | 3.63 | 117.70 | 68.64 |
| 2030 | 179.59 | 122.72 | 20.49 | 12.89 | 6.34 | 3.63 | 117.71 | 68.64 |
| 2031 | 180.31 | 123.24 | 20.54 | 12.93 | 6.33 | 3.63 | 117.73 | 68.65 |
| 2032 | 180.95 | 123.71 | 20.58 | 12.96 | 6.32 | 3.63 | 117.74 | 68.65 |
| 2033 | 181.53 | 124.14 | 20.62 | 12.98 | 6.31 | 3.63 | 117.75 | 68.65 |
| 2034 | 182.06 | 124.52 | 20.66 | 13.01 | 6.30 | 3.63 | 117.77 | 68.66 |
| 2035 | 182.53 | 124.86 | 20.69 | 13.03 | 6.29 | 3.63 | 117.78 | 68.66 |
| 2036 | 182.95 | 125.17 | 20.72 | 13.05 | 6.29 | 3.63 | 117.79 | 68.66 |
| 2037 | 183.33 | 125.45 | 20.75 | 13.07 | 6.28 | 3.63 | 117.79 | 68.67 |
| 2038 | 183.68 | 125.70 | 20.77 | 13.09 | 6.28 | 3.63 | 117.80 | 68.67 |
| 2039 | 183.98 | 125.92 | 20.79 | 13.10 | 6.27 | 3.63 | 117.81 | 68.67 |
| 2040 | 184.26 | 126.13 | 20.81 | 13.11 | 6.27 | 3.63 | 117.81 | 68.67 |
| 2041 | 184.51 | 126.31 | 20.83 | 13.13 | 6.26 | 3.63 | 117.82 | 68.68 |
| 2042 | 184.74 | 126.47 | 20.84 | 13.14 | 6.26 | 3.63 | 117.82 | 68.68 |
| 2043 | 184.94 | 126.62 | 20.86 | 13.15 | 6.26 | 3.63 | 117.83 | 68.68 |
| 2044 | 185.12 | 126.75 | 20.87 | 13.15 | 6.26 | 3.63 | 117.83 | 68.68 |
| 2045 | 185.29 | 126.87 | 20.88 | 13.16 | 6.25 | 3.63 | 117.84 | 68.68 |
| 2046 | 185.43 | 126.98 | 20.89 | 13.17 | 6.25 | 3.63 | 117.84 | 68.68 |
| 2047 | 185.57 | 127.08 | 20.90 | 13.18 | 6.25 | 3.63 | 117.84 | 68.68 |
| 2048 | 185.69 | 127.17 | 20.91 | 13.18 | 6.25 | 3.63 | 117.84 | 68.68 |
| 2049 | 185.80 | 127.24 | 20.91 | 13.19 | 6.25 | 3.63 | 117.85 | 68.68 |
| 2050 | 185.89 | 127.32 | 20.92 | 13.19 | 6.24 | 3.63 | 117.85 | 68.68 |
